# Acupoint catgut embedding alleviates experimental autoimmune encephalomyelitis by modulating neuroinflammation and potentially inhibiting glia activation through JNK and ERK pathways

**DOI:** 10.3389/fnins.2024.1520092

**Published:** 2025-01-09

**Authors:** Xiaofang Liu, Liansheng Yang, Zhumin Su, Xueying Ma, Yingying Liu, Lili Ma, Xiaomeng Ma, Mingxia Ma, Xiaoyun Liu, Kun Zhang, Xiaohong Chen

**Affiliations:** ^1^Department of Neurology, The Third Affiliated Hospital of Sun Yat-Sen University, Guangzhou, China; ^2^Department of Acupuncture and Moxibustion, The Third Affiliated Hospital of Sun Yat-sen University, Guangzhou, China; ^3^Department of Cardiology, Shanxi Province Cardiovascular Hospital, Taiyuan, China; ^4^Department of General Medicine, The Third Affiliated Hospital of Sun Yat-sen University, Guangzhou, China; ^5^Department of Allergy, The Third Affiliated Hospital of Sun Yat-sen University, Guangzhou, China

**Keywords:** acupoint catgut embedding, experimental autoimmune encephalomyelitis, microglia, astrocytes, inflammatory cytokine

## Abstract

**Background:**

Acupoint catgut embedding (ACE) is a traditional Chinese medicine technique commonly used for managing various disorders, including chronic inflammatory pain and allergic asthma. Despite its growing use, the neuroimmunological mechanisms underlying ACE treatment effects remain unclear.

**Methods:**

This study investigated the roles and potential mechanisms of the effects of ACE in treating experimental autoimmune encephalomyelitis (EAE), a frequently used animal model of autoimmune neuroinflammation. The effects of ACE treatment were evaluated by monitoring body weight and EAE severity scores. Behavioral tests, histopathological analysis, ELISA, and flow cytometry were conducted to assess the therapeutic efficacy of ACE. RNA sequencing was performed to uncover ACE-associated transcriptional signatures in the spinal cords of EAE mice.

**Results:**

The results were validated through western blotting, qRT-PCR, and immunofluorescence (IF) staining. In ACE-treated mice, EAE disease severity was significantly ameliorated, along with improvements in anxiety-like behaviors and reduced inflammation and demyelination. The ACE treatment restored immune imbalance in the EAE mice by decreasing Th17 and Th1 cells, while increasing Treg cells in peripheral immune organs and reducing serum inflammatory cytokine levels. RNA sequencing revealed significant suppression of the genes and pathways associated with reactive microglial and astrocytic activation, corroborated by IF studies. Additionally, ACE treatment could suppress the ERK and JNK signaling pathways at both RNA and protein levels.

**Conclusion:**

These findings confirm the protective role of ACE in mitigating EAE symptoms by modulating microglial and astrocytic activity and regulating inflammatory cytokines.

## Graphical abstract

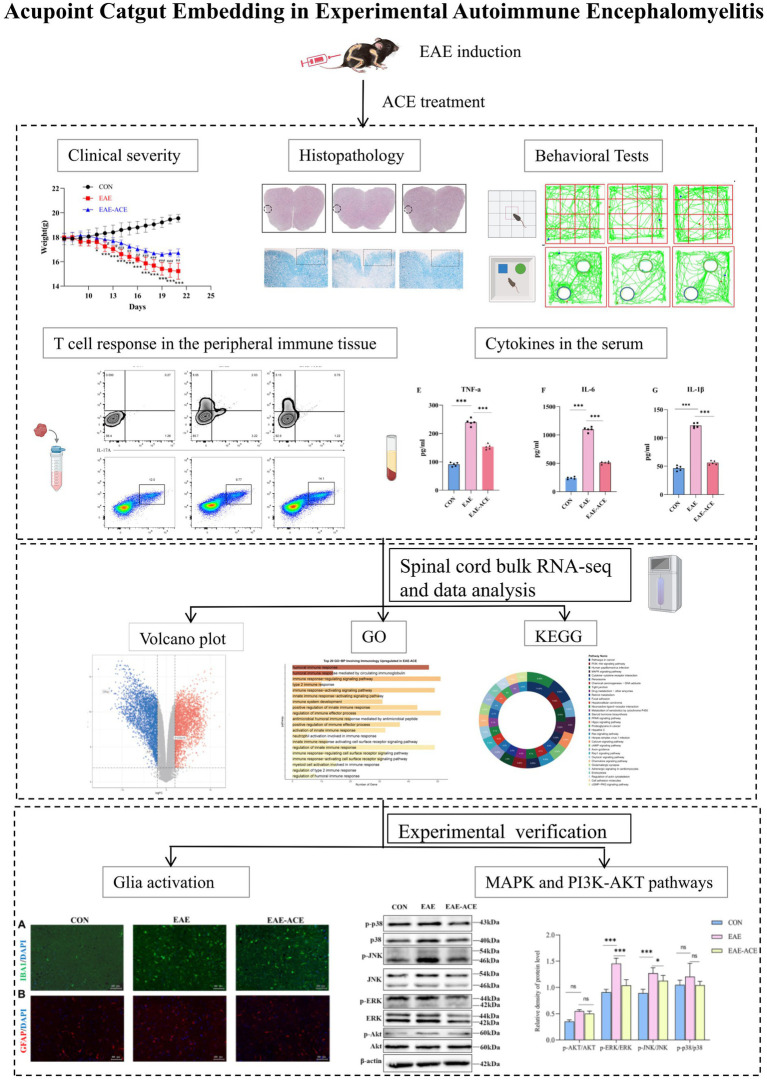

## 1 Introduction

Multiple sclerosis (MS) is a chronic inflammatory demyelinating disease of the central nervous system (CNS) that affects the brain (white matter), spinal cord, brainstem, and cerebellum. It manifests as a spectrum of clinical symptoms, including motor and sensory dysfunctions, as well as psychiatric disturbances (Koutsouraki et al., 2023). MS development is attributed to complex interactions between the immune system, neurons, and glial cells (Goris et al., 2022). Current MS treatments primarily involve systemic immunosuppressive or immunomodulatory drugs. However, a subset of patients respond poorly or experiences severe adverse effects (Mansilla et al., 2021). Consequently, a deeper understanding of MS pathogenesis and exploring novel therapeutic approaches are crucial.

Traditional Chinese medicine (TCM), including herbal medicine, acupuncture, and Chinese therapeutic massage, has garnered increasing attention for its multi-target and holistic therapeutic effects against many human diseases. Acupuncture, one of the most widely used TCM modalities, aims to restore bodily homeostasis by stimulating specific acupoints to activate self-healing mechanisms. Emerging evidence highlights acupuncture’s efficacy in managing various immunological disorders, such as sepsis (Liu et al., 2021), inflammatory bowel syndrome (Manheimer et al., 2012), and allergic asthma (Tang et al., 2021). In MS, acupuncture has been shown to efficiently alleviate neurological impairments, reduce fatigue, and improve patient’s quality of life (Donnellan and Shanley, 2008; Wang C. et al., 2017). Electroacupuncture (EA), a variation involving electrical stimulation of acupoints such as Zusanli (ST36), has demonstrated significant therapeutic effects. For instance, EA mitigates allergic skin inflammation by inhibiting Th1 differentiation, restoring Th1/Th2 balance, promoting IL-10 production, and suppressing p38 MAPK activation. This results in the amelioration of Th1-mediated allergic dermatitis (Wang et al., 2017a,b). In models of inflammatory pain, EA alleviates postoperative immunosuppression by reducing T-cell infiltration and IL-6 expression. Moreover, it inhibits glial cell activation by modulating chemokines such as CX3CL1 and increasing anti-inflammatory cytokines such as IL-10 while suppressing proinflammatory mediators like TNF-α, IL-1β, IL-6, and prostaglandin E2 (Fu et al., 2007; Li et al., 2019; Song et al., 2023). Studies have further shown that EA at ST36 alleviates experimental autoimmune encephalomyelitis (EAE), the MS animal model, by reducing Th1/Th17 cell proportions, increasing Th2 cell proportions, and mitigating CNS inflammatory cell infiltration and demyelination (Wang et al., 2017a; Zhao et al., 2021). However, the daily administration required for EA presents practical challenges for both clinicians and patients.

Beyond EA, the primary methods of acupuncture therapy include manual acupuncture. However, both traditional approaches have limitations, such as short stimulation durations and the need for frequent clinic visits. Acupoint catgut embedding (ACE), a novel “Acupuncture+” strategy, represents an innovative extension of traditional acupuncture (Chen et al., 2020). Unlike classic acupuncture methods, ACE involves embedding absorbable suture segments into acupoints. This provides prolonged stimulation, allowing the body to experience sustained physiological effects over an extended period. ACE exerts immunoregulatory effects by inhibiting inflammatory factor generation (Yang et al., 2018; Teng et al., 2022). The embedded catgut serves as a durable stimulus within the specific acupoint, gradually undergoing softening, liquefaction, and absorption due to combined chemical and mechanical stimulation. Thus, ACE effectively integrates traditional acupuncture principles with modern tissue treatment. Clinically, ACE is primarily used to address conditions such as weight management, insomnia, inflammatory pain, and rheumatoid arthritis (Garcia-Vivas et al., 2014; Guo et al., 2015; Du et al., 2017). Notably, ACE has demonstrated superior outcomes compared with manual acupuncture and shows greater effectiveness in weight loss compared with EA (Guo et al., 2015). Despite its growing use, the mechanisms through which ACE protects against CNS autoimmune diseases remain poorly understood.

Guided by the meridian system and traditional acupuncture principles such as “like cures like” and “treating the root and branch” (Wang C. et al., 2017), three acupoints were selected for this study: Zusanli (ST36), Quchi (LI11), and Shenshu (BL23). Zusanli (ST36) is rich in nerves and blood vessels. A study published in *Nature Medicine* (2014) demonstrated that stimulating Zusanli activates dopamine release, facilitating vagus nerve regulation of the immune system (Torres-Rosas et al., 2014). Additionally, Professor Qiufu Ma’s team revealed in 2021 that EA at Zusanli exerts anti-inflammatory effects through the vagus nerve-adrenal axis (Liu et al., 2021). Anatomically corresponding to the mouse kidney, Shenshu (BL23) is associated with neurohumoral regulatory responses, improving local tissue metabolism and overall physiological balance (Zhu et al., 2024). Quchi (LI11) is linked to meridian qi and blood circulation in the upper limbs. Its stimulation regulates the immune function, promotes neuroprotection, and helps regulate systemic immunity (Tao et al., 2016). This study used a myelin oligodendrocyte glycoprotein 35–55 (MOG35–55)-induced EAE model to explore the effects and mechanisms of ACE in treating CNS autoimmune diseases. By targeting EAE, a widely recognized animal model for MS, we investigated the therapeutic efficacy of ACE and its underlying mechanisms. The results can provide an understanding on how ACE therapy alleviates EAE, thus highlighting its potential in treating CNS disorders and promoting the application of TCM in the treatment of such diseases.

## 2 Materials and methods

### 2.1 Animals

The experiments were conducted using 6- to 8-week-old female C57BL/6 mice. After a 1-week acclimatization period, the mice were randomized to three groups (*n* = 6 per group): control, EAE, and ACE-treated EAE (EAE-ACE) groups. The Animal Care and Use Committee of South China Agricultural University (Approval ID: 2022D157) approved all experimental protocols in compliance with the ARRIVE guidelines and the *Guide for the Care and Use of Laboratory Animals*.

### 2.2 EAE induction

EAE was induced in the EAE and EAE-ACE groups through active immunization at upper/lower back (100 μL emulsion/site). The mice were subcutaneously injected with 200 μg MOG35–55 emulsified in 200 μL of complete Freund’s adjuvant (CFA, Sigma F5881, United States), containing 5 mg/mL of the *Mycobacterium tuberculosis* strain H37Ra (BD 231141, United States). The emulsion was prepared using phosphate-buffered saline (PBS) under sterile conditions. Additionally, 250 ng pertussis toxin (PTX181, List Biological Laboratories, United States) was administered intraperitoneally on the day of immunization and 2 days later. A booster injection of MOG35–55 emulsified in CFA was delivered 7 days after the initial injection (Lin et al., 2020). The mice were weighed daily, and EAE clinical symptoms were monitored by an experimenter using the following grading scale: grade 5, death; grade 4.5, close to death or moribund; grade 4, total bilateral limb paralysis; grade 3, total unilateral limb paralysis; grade 2.5, partial limb paralysis and ataxia; grade 2, gait dysfunction with ataxia and limp tail; and grade 1, gait dysfunction with limp tail or reduced tail tone. Intermediate clinical signs were scored in increments of 0.5 as necessary (Yu et al., 2023).

### 2.3 ACE therapy

**Figure 1:**
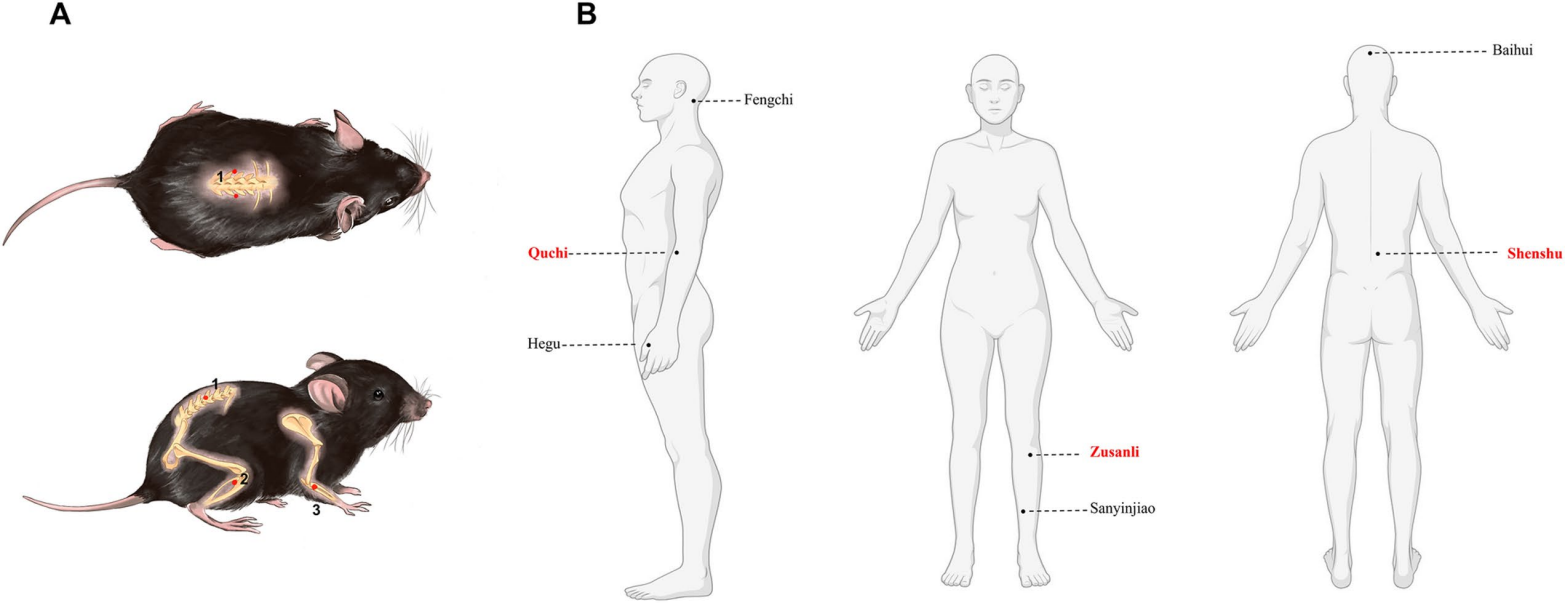
Mouse and human maps of acupoints used in MS. **(A)** Acupoint diagram of ACE in C57BL/6 mouse. (1) Shenshu (BL23). (2) Quchi (LI11). (3) Zusanli (ST36). **(B)** Maps of acupoints used commonly in patients with multiple sclerosis.

The mice in the EAE-ACE group underwent ACE treatment on days 7 and 14 post-immunization (dpi). The treatment was administered using disposable embedding needles (diameter, 0.7 mm; Gaoguan Medical, Zhenjiang, China) and absorbable catgut (collagen threads, 0.5 cm length, 2–0 grade; 2 cm × 10, BD150101, Boda Co., Ltd., Shandong, China). Prior to thread embedding, sterile No. 4-0 collagen threads were cut into 5-mm pieces and sterilized in normal saline. The mouse skin surrounding the acupoints was disinfected with 75% alcohol. The specific acupoint was fixed using the thumb and index finger, and a needle was inserted at a 45° angle toward the spine, penetrating to a depth of 0.3 cm. Once the acupoint was reached, the needle core was pushed, followed by the withdrawal of the needle tube and embedding of the collagen thread into subcutaneous and muscle layers (Teng et al., 2022). The treated acupoints were Shenshu (BL23), Quchi (LI11), and Zusanli (ST36). Shenshu (BL23) is located below the second lumbar vertebrae, 3 mm lateral to the midline of the back. Quchi (LI11) is located in the depression on the anterior-lateral aspect of the proximal radius near the elbow joint. Zusanli (ST36) is located posterolateral to the knee joint, approximately 2 mm below the fibular head. “Common acupuncture points and their location in laboratory animals part 3: mice” can be found in detail in the Appendix. Figure 1 displays a schematic of the acupoint locations. Figure 1B was created using assets from BioRender, and the relevant usage permission can be found in the Supplementary material.^1^ The EAE group underwent a sham-ACE procedure, following the same steps but without embedding the collagen thread. The detailed ACE procedure in shown in Supplementary Figure S1.

### 2.4 Behavior tests

The mice were allowed to acclimatize to the testing room for 1 h prior to each behavior test.

#### 2.4.1 Open-field test

The mice were placed in a white, non-transparent plastic open-field arena (40 cm × 40 cm × 25 cm) divided into 16 zones: an outer zone consisting of 12 peripheral zones and an inner zone comprising four central zones (Choleris et al., 2001). The mice were allowed 5 min of free exploration, followed by 10 min of tracked movement recorded using SuperMaze software (Shanghai Xinruan Information Technology Co., Ltd., Shanghai, China). The behavioral parameters analyzed included total distance moved (cm); number of entries into the inner zone; total time spent in the inner zone (s), and time spent in the outer zone (s). The open-field test (OFT) was conducted to assess motor function and anxiety-like behavior. Mice spending more time at the edges of the box and less time in the center were considered to exhibit higher anxiety-like behavior (Yamanoi et al., 2019). Following every test, the arena was cleaned with 75% ethanol to eliminate olfactory cues in the box.

#### 2.4.2 Novel object recognition test

The novel object recognition test (NORT) (Li Q. et al., 2024), following a previous method, was conducted in a white open-field observation box (40 cm × 40 cm × 25 cm). This test comprised two phases. In Phase 1 (T1), two identical objects were symmetrically placed at equidistant points along the diagonal of the box floor. The mice were introduced into the observation box for a 10-min free exploration session, during which their interactions with the objects were recorded. Afterward, the mice were returned to their respective cages.

Following a 1-h interval, the second phase (T2) began. One of the original objects was substituted by a novel object, differing in shape and color. The mice were reintroduced into the observation box for another 10-min exploration period. A video analysis system recorded movement trajectories, speeds, and exploration times for each object. The exploration times for the familiar object (FT) and the novel object (NT) were measured. The discrimination ratio (DR) was calculated using the formula: DR = [NT/(NT + FT)] × 100%.

### 2.5 Histopathology

On 21 dpi, the lumbar spinal cords were dissected from mice in the control, EAE, and EAE-ACE groups for histopathological analysis. The mice were fixed by cardiac perfusion with 4% (w/v) paraformaldehyde. The spinal cords were then extracted, paraffin-embedded, sectioned, and stained with hematoxylin-eosin (HE) and luxol fast blue (LFB) to evaluate inflammatory cell infiltration and demyelination, respectively. Inflammatory cell infiltration was scored as follows: 0 was considered to indicate the absence of inflammatory cells; (1) suggested the presence of several scattered inflammatory cells, (2) indicated inflammatory infiltrate organization surrounding blood vessels, and 3 suggested excess perivascular cuffing extending into adjacent parenchyma and parenchymal infiltration with no significant cuffing. In addition, demyelination was scored as follows: (1) representing traces of subpial demyelination; (2) suggesting evident subpial/perivascular demyelination; (3) indicating confluent perivascular/subpial demyelination; (4) representing extensive demyelination involving half the spinal cord with cellular infiltration into the CNS parenchyma; (5) demonstrating extensive perivascular/subpial demyelination involving the entire spinal cord section with infiltration into the CNS parenchyma (Xie et al., 2023).

### 2.6 Nissl staining

Paraffin sections were deparaffinized and rehydrated before immersion in toluidine blue staining solution for 5 min. Afterward, the sections were rinsed with water and mildly differentiated with 1% acetic acid. The reaction was terminated with tap water. The sections were dried in an oven, rendered transparent following treatment with xylene for 5 min, and mounted using neutral balsam. Cortical neurons in the stained sections were observed under a microscope by using CaseViewer software (Li P. et al., 2024). Neuronal damage was assessed by counting Nissl-stained positive cells in the cortex under a 50 μm scale. Three random fields per section were analyzed using ImageJ software to compare the degree of cortical neuronal damage among the different groups.

### 2.7 ELISA

Serum samples were collected aseptically from mice on 21 dpi and immediately stored at −80°C. Later, TNF-a, IL-1b, and IL-6 concentrations were measured using TNF-a (Invitrogen, BMS607-3, United States), IL-1β (EMC001b.96), and IL-6 (EMC004.96) (Neobioscience, China) ELISA kits, respectively, according to the manufacturer’s specific guidelines.

### 2.8 Flow cytometry

Spleens and inguinal lymph nodes (ILNs) were harvested from the treated mice for flow cytometry analysis. The cells were fixed, permeabilized (Chen et al., 2017), and stained with fluorophore-conjugated antibodies targeting intracellular cytokines and nuclear transcription factors, separately. The samples were analyzed using a CytoFLEX flow cytometer, and data were processed using FlowJo software (Tree Star, Ashland, OR). Specific panel configurations are provided in Supplementary Table S1.

### 2.9 Bulk RNA-seq and data processing

TRIzol reagent (Invitrogen Life Technologies, Carlsbad, CA, United States) was used for extracting total RNA from the spinal cord tissue. Thereafter, RNA quality and quantity were evaluated using RseQC 2.4 software, and purification was performed twice using AMPure XP beads. Subsequently, cDNA was prepared from RNA through reverse transcription by using the mRNASeq sample synthesis kit (Illumina, San Diego, CA, United States). cDNAs were sequenced using the Illumina NovaseqTM 6000 platform. Differentially expressed genes (DEGs) were identified using the DESeq2 R-package, with selection criteria of log2 fold change >2 and *p* value <0.05. Gene Ontology (GO) and Kyoto Encyclopedia of Genes and Genomes (KEGG) enrichment analyses were performed on the DEGs,^2^ with *p* < 0.05 representing statistical significance.

### 2.10 Immunofluorescence analysis

Paraffin-embedded mouse brain cortex and spinal cord tissues were cut into 20-μm slices. The slices were then deparaffinized in xylene and rehydrated with gradient ethanol concentrations. For antigen retrieval, the sections were heated in 10 mM citrate buffer (pH 6.0) for 10 min by using a microwave oven, followed by cooling for 30 min at room temperature. Non-specific binding was blocked with 5% bovine serum albumin (BSA) in PBS containing 0.3% Triton X-100 for 1 h at room temperature. The sections were incubated overnight with the primary antibodies (1% BSA in PBS) at 4°C. The primary antibodies included rabbit anti-Iba1 (1,200, Abcam) and mouse anti-GFAP (1,5,000, CST). After three washes with PBS, the sections were incubated with secondary antibodies (FITC-conjugated goat anti-rabbit IgG and Cy3-conjugated goat anti-mouse IgG, both at 1:400; Servicebio) for 1 h.

Images spanning the full slice thickness were captured using the TissueFAXS platform (TissueGnostics, Vienna, Austria) (20× magnification) and analyzed using ImageJ software (Fiji edition, NIH). Image analysis was completed by a blinded researcher.

### 2.11 Western blotting

Spinal cord samples were resolved on 12.5% sodium dodecyl sulfate-polyacrylamide gels (30 μg protein/lane) and transferred onto PVDF membranes. The membranes were blocked with 5% defatted milk and incubated overnight with primary antibodies against p-p38 (1:2,000; CST, 9,211), p38 (1:2,000;CST, 9,212), p-JNK (1:2,000;CST, 58,328), JNK (1:2,000; CST, 9,252), p-ERK (1:1,000; CST, 4,370), ERK (1:1,000; CST, 4,695), p-Akt (1:1,000; CST, 4,060), and Akt (1,1,000; CST, 4,691). After the membranes were washed with TBST buffer three times, they were incubated with goat anti-rabbit HRP-conjugated secondary antibodies for additional 30 min. Western blot assays were performed in triplicate, with GAPDH used as the loading control. Band intensities were quantified using Image J software.

### 2.12 qRT-PCR

VeZol Reagent (Vazyme) was used to extract RNA. The extracted RNA was reverse-transcribed into cDNA by using the HiScript III RT SuperMix for qPCR (+gDNA wiper) Transcription Kit (Vazyme). Gene expression levels were quantified through qPCR with SYBR Green Mix (Vazyme), normalized to GAPDH, and compared with those in the control group. Supplementary Table S2 presents the sequences of primers used.

### 2.13 Statistical analysis

Data are presented as the mean ± SEM from at least three independent experiments. Statistical analyses were conducted using GraphPad Prism 9.0. Between-group comparisons were performed using Student’s *t*-test, whereas multiple group comparisons were assessed using one-way and two-way ANOVA with Bonferroni post-tests. Specific tests are provided in figure legends. Statistical significance was set at *p* < 0.05.

## 3 Results

### 3.1 ACE mitigated clinical severity and neurobehavioral impairments in EAE mice

**Figure 2:**
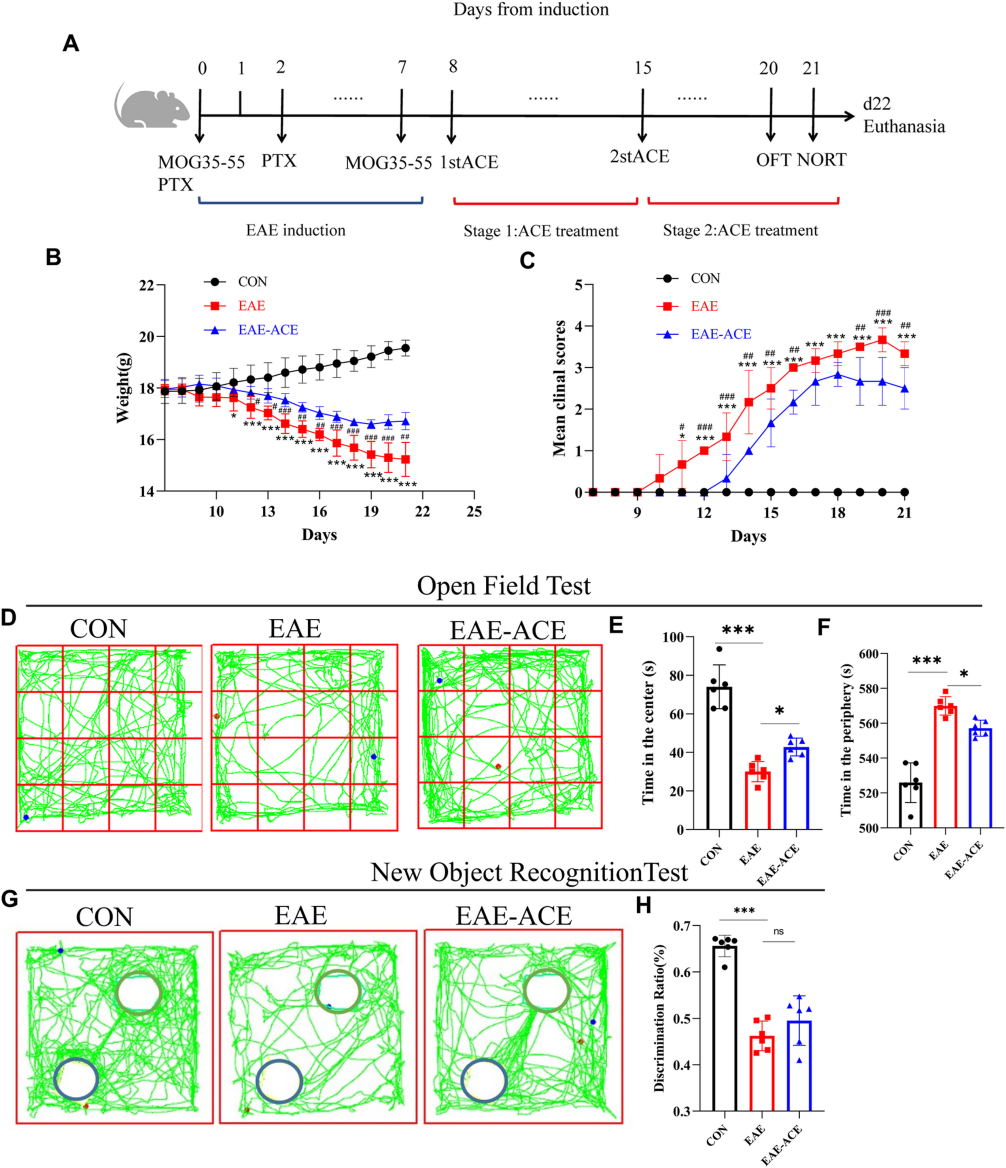
Treated EAE mice showed decreased disease scores and neurobehavioral impairments. **(A)** Schematic showing the timeline for EAE induction, ACE treatment, and behavior experiments. **(B)** The mean weight of mice in the control, EAE, and EAE-ACE groups. **(C)** Mean disease scores of mice in the control, EAE, and EAE-ACE groups. **(D)** Mouse movement trajectory graph in the OFT. **(E)** Time in the center decreased in EAE mice but increased significantly after ACE treatment. **(F)** Time in the periphery was in direct contrast to this observation. **(G)** Mouse movement trajectory graph in the NORT. The blue circle represents the new object, while the green circle represents a familiar object. **(H)** In NORT, the discrimination ratio (*p* < 0.001) decreased in the EAE mice but increased in the EAE-ACE mice (*p* = 0.3228). Significance is marked by asterisk (*n* = 6 per group; two-way ANOVA with Bonferroni’s post-tests; ^*^p < 0.05, ^**^p < 0.01, and ^***^p < 0.001).

To examine the impact of ACEs on CNS autoimmune diseases, an EAE animal model was established, and disease severity of the mice was assessed following ACE treatment. Weight loss in EAE mice began around day 12 post-immunization, while motor deficits first appeared on day 10 after immunization (Figures 2B,C). As displayed in the experimental timeline (Figure 2A), ACE treatment delayed disease onset, reduced symptom severity, and slowed weight loss compared with the control. EAE is characterized by not only motor impairments but also higher neurological dysfunctions, such as cognitive deficits and mood disturbances, including depression-/anxiety-like behaviors (Acharjee et al., 2013). In the OFT, depressive states are associated with significantly reduced total movement distance, while anxiety is indicated by a decreased frequency of entries into the central zone and reduced time spent in the center, with a corresponding increase in time spent at the periphery (Choleris et al., 2001; Fatemi et al., 2016). In our study, EAE mice exhibited a marked reduction in total movement distance, potentially due to motor dysfunction or weight changes (Figure 2D). ACE-treated mice showed an improvement in total movement distance (Supplementary Figure S2A). However, compared with the untreated EAE mice, ACE-treated mice displayed a slight reduction in entries into the central zone (Supplementary Figure S2B) and time spent in the center (Figure 2E), with an increase in time spent at the periphery (Figure 2F). These findings indicated that while ACE treatment partially alleviates anxiety-like behaviors in EAE mice, residual clinical deficits and motor impairments remain. Cognitive function, assessed through the NORT, was significantly impaired in the EAE mice. Although the DR increased with ACE treatment, the improvement was not statistically significant (Figures 2G,H).

### 3.2 ACE alleviated histopathological changes in EAE mice

**Figure 3:**
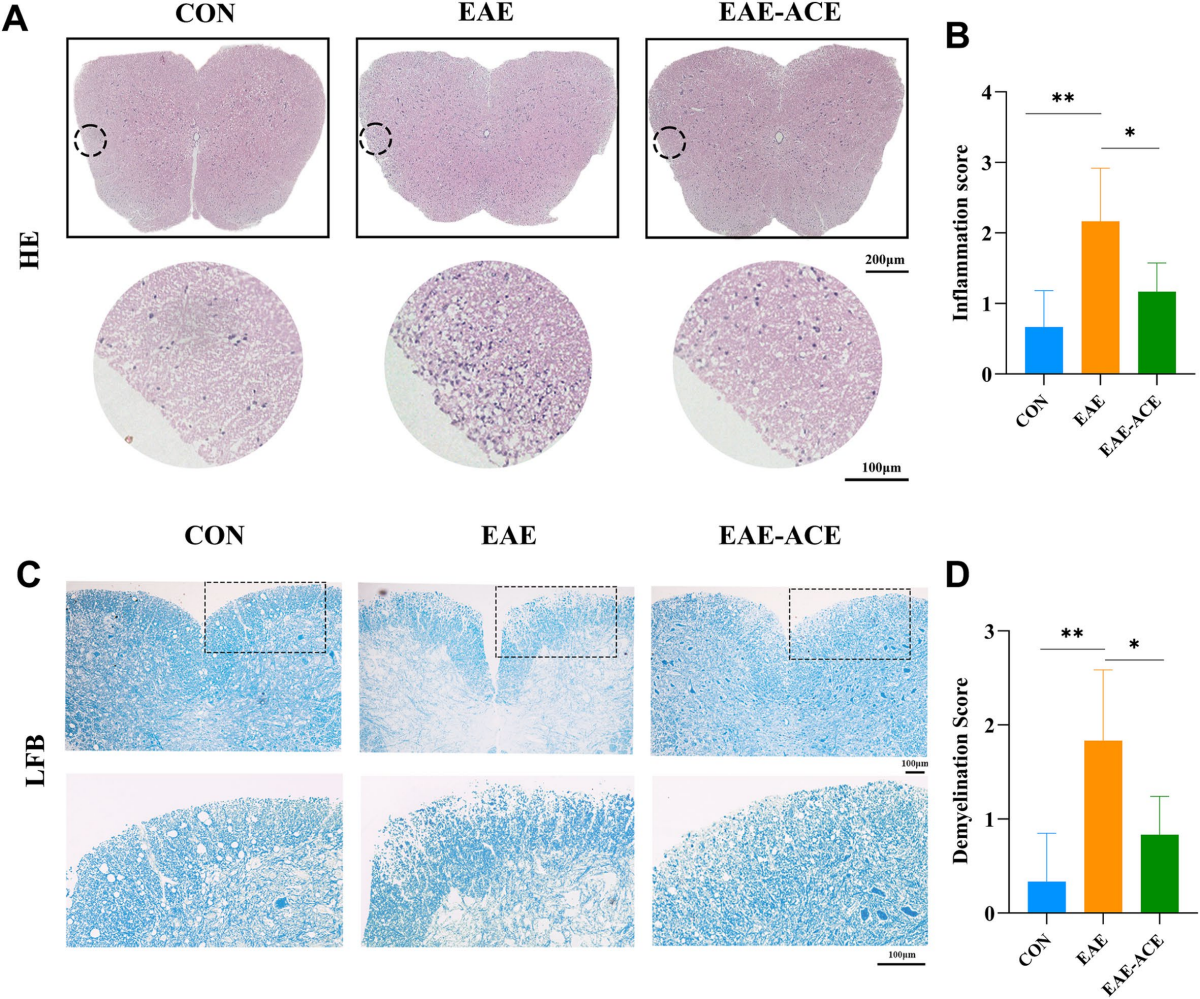
ACE treatment significantly decreased inflammation and demyelination in the spinal cords of EAE mice. At 21 dpi, lumbar spinal cords were isolated and subjected to HE staining **(A)** and LFB staining **(C)** to evaluate histopathological changes. Representative sections **(A,C)** and corresponding statistical analyses **(B,D)** are presented. Each group consisted of six mice (*n* = 6). Data are expressed as the mean ± SEM. Statistical significance was determined using one-way ANOVA with Bonferroni’s post-tests (^*^p < 0.05 and ^**^p < 0.01).

To investigate whether ACE mitigates CNS inflammation and demyelination, a histopathological analysis was conducted on lumbar spinal cord samples. The results demonstrated a significant reduction in inflammatory cell infiltration scores in the white matter of the lumbar spinal cord in ACE-treated EAE mice compared with the untreated EAE mice (Figures 3A,B). Extensive demyelination plaques were observed in the lumbar spinal cords of vehicle-treated EAE mice, whereas these plaques were significantly reduced in the ACE-treated group (Figures 3C,D). Additionally, to assess neuronal damage, Nissl staining was performed on mouse brain samples. However, no significant difference in the number of Nissl-stained neurons was observed between the untreated EAE group and the ACE-treated group (Supplementary Figure S3).

### 3.3 ACE modulates peripheral response in CD4^+^ T cells and inflammatory cytokines in EAE mice

**Figure 4:**
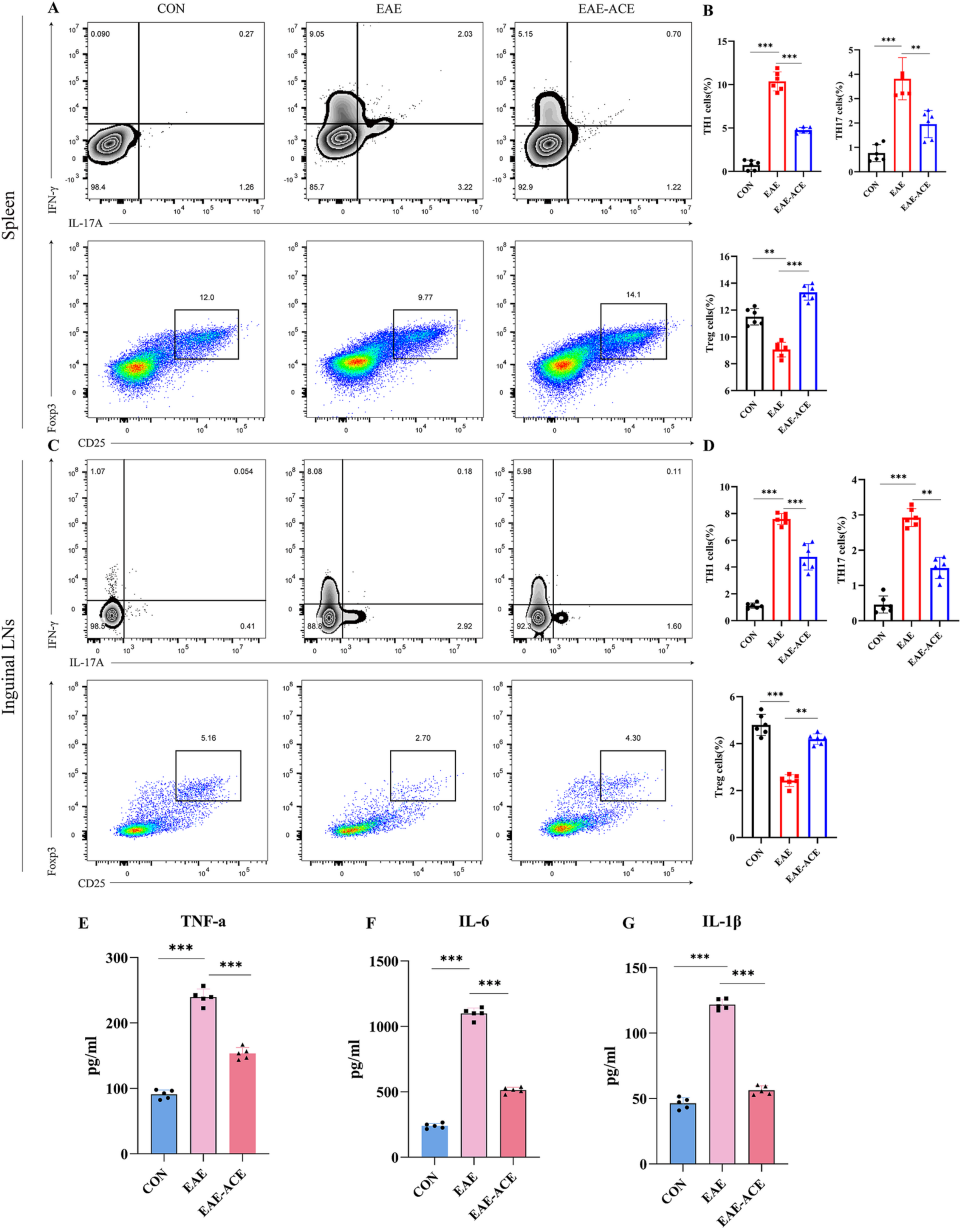
ACE modulated T-cell response in the spleen and ILNs and reduced inflammatory cytokines in serum of the EAE mouse. Mononuclear cells were harvested from the spleen and ILNs of all mice at 21 dpi. and subsequently analyzed through flow cytometry. Mononuclear cells were first gated for lymphocyte, followed by the gating of CD4^+^ T cells and then analyzed for the percentages of Th17 (CD4^+^ IL-17A^+^), Th1 (CD4^+^ IFNγ^+^), and Treg (CD4^+^ CD25^+^ Foxp3^+^) cells. Th17, Th1, and Treg cells in the spleen and statistical analysis **(A–D)** (*n* = 6 in each group). Sera of all mice were aseptically harvested at 21 dpi. Serum was analyzed through ELISA to determine the levels of IL-1β **(E)**, IL-6 **(F)**, and TNF-α **(G)** (*n* = 5). The results are presented as the mean ± SEM. ^*^p < 0.05, ^**^p < 0.01, and ^***^p < 0.001, one-way ANOVA with Bonferroni’s post-tests.

The histopathological results demonstrated that the ACE treatment reduced CNS inflammatory infiltration in the EAE mice. CD4^+^ T cells, including their subpopulations Th1, Th17, and Treg cells, are well-recognized for their critical roles in MS/EAE pathogenesis. Th1 and Th17 cells are generally considered pathogenic, while Treg cells exhibit protective properties (Jäger et al., 2009; Lin et al., 2020). To explore the effects of ACE on CD4^+^ T-cell differentiation in EAE mice, we conducted flow cytometry to quantify Th1 (CD4^+^ IFN-γ^+^), Th17 (CD4^+^ IL-17^+^), and Treg (CD4^+^ CD25^+^ Foxp3^+^) populations in the spleen and ILNs. Our results revealed a significant increase in the Th1 and Th17 cell proportions and a notable decrease in the Treg cell proportions in the spleen and ILNs of EAE mice. However, ACE treatment reversed this trend, significantly reducing Th1 and Th17 cells and increasing Treg cells in the EAE-ACE group compared with those in the untreated EAE group (Figures 4A–D).

To further investigate the systemic inflammatory response, we measured the serum levels of key proinflammatory cytokines—TNF-α, IL-6, and IL-1β—using ELISA. These cytokines are known to contribute significantly to the MS/EAE pathogenic mechanism. EAE mice exhibited elevated serum levels of TNF-α, IL-6, and IL-1β, whereas ACE treatment significantly reduced the serum levels of these cytokines in EAE mice (Figures 4E–G).

### 3.4 ACE altered transcriptomic signatures in the spinal cord, targeting immune and signaling pathways in EAE mice

**Figure 5:**
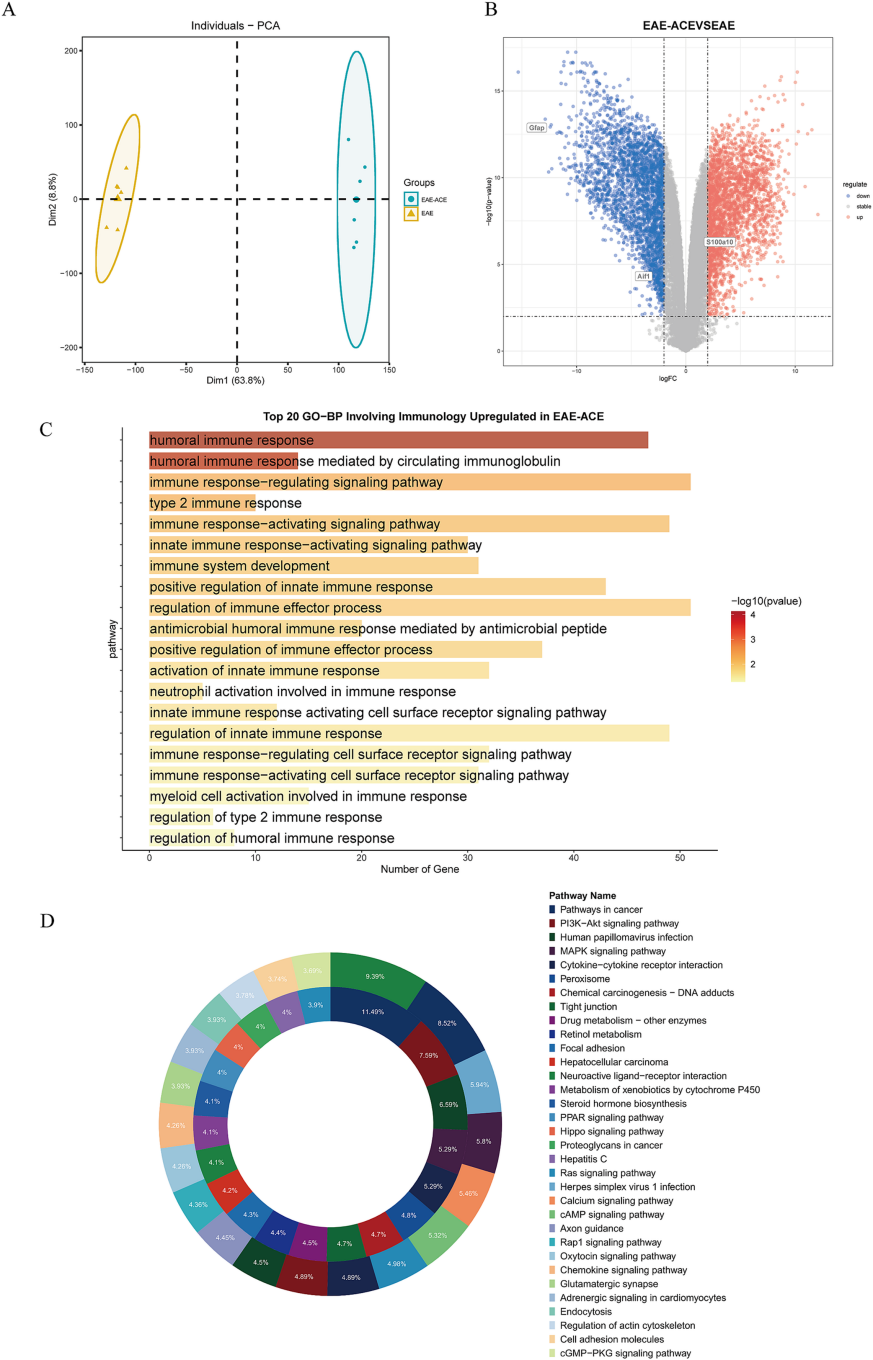
Transcriptome analysis on spinal cords from the mice in EAE and EAE-ACE groups. **(A)** Principal component analysis was conducted for the EAE and EAE-ACE groups based on the expression patterns. **(B)** Volcano plot of spinal cord RNA-seq analysis of EAE-ACE vs. EAE. **(C)** Top 20 upregulated biological processes involving the immune response and **(D)** KEGG analysis of the upregulated and downregulated DEGs in the EAE-ACE and EAE groups.

To elucidate the transcriptional alteration induced by ACE treatment and the molecular mechanisms underlying ACE’s therapeutic effects on EAE, we performed RNA sequencing of spinal cord tissues from three groups: control, EAE, and EAE-ACE. Principal component analysis (PCA) revealed distinct transcriptomic profiles between the EAE and EAE-ACE groups, indicating substantial transcriptional reprogramming induced by ACE treatment (Figure 5A). Differential gene expression analysis identified 9,127 DEGs in the EAE-ACE group compared with the EAE group, comprising 2,694 downregulated and 6,433 upregulated genes (log2 FC >2 and *p* < 0.05, Figure 5B). GO analysis demonstrated that the DEGs were significantly enriched in immune-related functions, highlighting ACE’s impact on modulating immune responses (Figure 5C). The KEGG pathway analysis further revealed that the DEGs were associated with several critical signaling pathways, including the PI3K-AKT and MAPK pathways, which are implicated in inflammation and neuroprotection (Figure 5D). These results indicated that ACE exerts its therapeutic effects by modulating the immune activity and key signaling pathways involved in EAE pathogenesis (Figure 5D).

### 3.5 ACE regulated glial activation and ERK and JNK signaling in EAE mice

**Figure 6:**
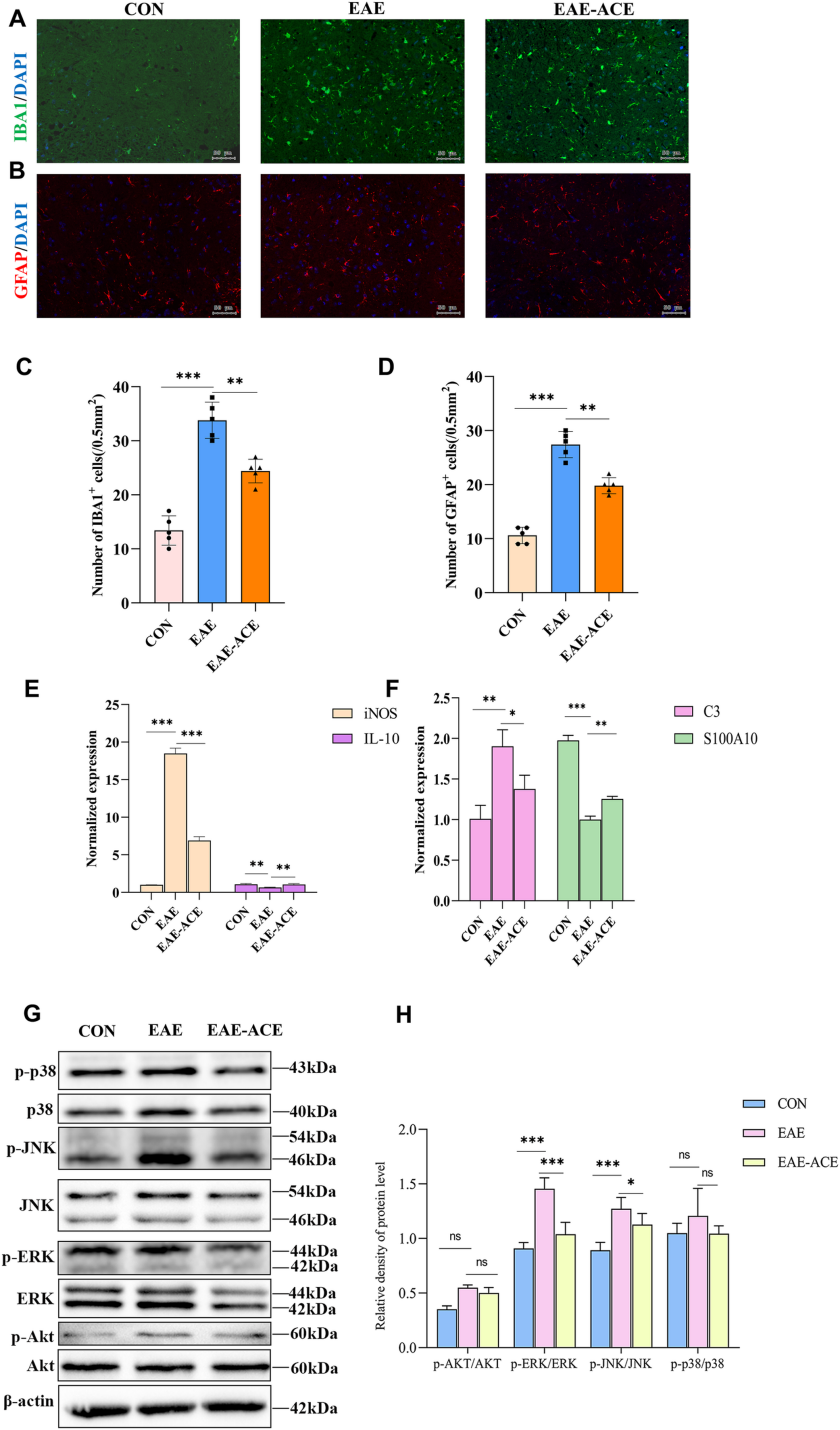
ACE treatment suppressed the activation of microglia and astrocytes and ERK and JNK pathways, while altering the expression of related proteins, in EAE mice. **(A,B)** Microglial reactivity, identified through Iba1 antibody staining, and astrocyte reactivity, identified through GFAP antibody staining, were assessed in the white matter of lumbar spinal cords. **(C,D)** IBA1^+^ and GFAP^+^ cells in the spinal cord were quantified using ImageJ software. (*n* = 6; scale bar, 50 μm). **(E)** qRT-PCR analysis of astrocyte-related genes, iNOS and IL-10, in the spinal cords of mice in the control, EAE, and EAE-ACE groups. **(F)** qRT-PCR analysis of astrocyte-related genes, C3 and S100A10, in the spinal cords of mice in the control, EAE, and EAE-ACE groups. Data are presented as the mean ± SEM. Statistical significance was determined using one-way ANOVA with Bonferroni’s post-tests (^*^p < 0.05, ^**^p < 0.01, and ^***^p < 0.001). **(G)** Western blot analysis for the expressions of p-p38, p38, p-JNK, JNK, p-ERK, ERK, p-Akt, and Akt in the lumbar spinal cords of the control, EAE, and EAE-ACE mice. **(H)** Western blot analysis and the densitometric quantification of p-p38, p38, p-JNK, JNK, p-ERK, ERK, p-Akt, and Akt in the spinal cord. The protein levels are expressed as percentages relative to the normal control. Each experiment was conducted on the samples pooled from three animals. The panel represents a typical result from three independent experiments. Data are presented as the mean ± SEM. Statistical significance was determined using one-way ANOVA with Bonferroni’s post-tests (*n* = 3, ^*^p < 0.05, ^**^p < 0.01, and ^***^p < 0.001).

Based on transcriptomic sequencing analysis, the beneficial effects of ACE in EAE mice appear to involve immune system regulation and inflammation-associated signaling pathways. Studies have also shown that ACE can modulate immune responses. We attempted to validate these findings at the cellular and protein levels. To assess glial activation, we evaluated changes in microglia and astrocytes within the lumbar spinal cord of the EAE mice through IF staining (Figures 6A–D). The EAE group exhibited increased numbers of IBA1^+^ microglia and GFAP^+^ astrocytes compared with controls. These findings aligned with the qRT-PCR results, which indicated that the levels of proinflammatory iNOS were significantly increased and those of anti-inflammatory IL-10 were reduced in the spinal cord of EAE mice. ACE treatment reduced iNOS expression and increased IL-10 levels, suggesting a shift from neurotoxic reactive microglia toward a more neuroprotective state (Figure 6E). Furthermore, ACE treatment modulated astrocytic activity by decreasing the level of the neurotoxic marker S100A10 and increasing the level of the neuroprotective marker C3 (Figure 6F). Additionally, in the cortex of EAE mice, ACE treatment could significantly reverse the increase in neurotoxic microglia and astrocytes observed in untreated animals (Supplementary Figure S4).

The MAPK signaling pathway plays a critical role in intracellular signaling transduction, with abnormal activation linked to the pathogenesis of inflammatory and neurodegenerative disorders. Key MAPK proteins include c-Jun N-terminal kinase (JNK), p38, and extracellular signal-regulated kinase (ERK) (Morrison, 2012). Similarly, the PI3K/Akt signaling pathway is integral to angiogenesis and inflammatory factor recruitment. PI3K activation leads to the phosphorylation of phosphatidylinositol-4,5-bisphosphate, thus forming phosphatidylinositol-3,4,5-trisphosphate, which subsequently activates downstream effectors, such as serine/threonine kinase Akt (Burke et al., 2023). Activated Akt can phosphorylate numerous substrates. As shown in Figures 6G,H, the phosphorylation levels of JNK, ERK, p38, and AKT in the spinal cord tissues of EAE mice were determined through western blotting. Compared with the control group, the EAE group exhibited markedly elevated levels of phosphorylated JNK and ERK proteins, whereas AKT and p38 exhibited a trend toward upregulation, with no statistical significance. ACE treatment remarkably reduced ERK and JNK phosphorylation, suggesting suppression of these pro-inflammatory pathways. However, no significant changes in the p38 and AKT signaling pathways were noted between the EAE and EAE-ACE groups. These observations suggested that ACE exerts its immunomodulatory effects possibly by suppressing the ERK and JNK pathways.

## 4 Discussion

This study demonstrates the beneficial effects of ACE on EAE mice, including improvements in disease severity, functional impairments, and reductions in the inflammatory cytokine levels. ACE treatment attenuated the proliferation of immunoreactive microglial cells and astrocytes in the CNS of EAE mice, potentially by modulating ERK and JNK pathways. These findings suggest that ACE serves as a potential therapeutic approach for managing MS.

ACE (specifically EA) is known to exert neuroprotective effects, such as alleviating first-episode major depressive disorder by activating the visual network (Wang et al., 2024) and reducing depressive-like behaviors in rats under chronic restraint stress by inhibiting TLR4 signaling pathway-induced neuroinflammation (Jiang et al., 2024). However, no study has specifically documented ACE’s ability to improve anxiety-like behaviors in mice. In this study, we observed decreased time spent in the center of the OFT among EAE mice, which could be attributed to both motor deficits and disrupted neuronal circuits—consistent with immune abnormalities observed in EAE. Neuroinflammation is known to influence anxiety-like behaviors in mice by reducing neurogenesis and causing neuronal loss (Peres et al., 2023). Our findings further suggest that the ACE treatment mitigates the production of inflammatory cytokines, such as IL-1β, IL-6, and TNF-α, and reduces immune cell infiltration. Therefore, more studies are warranted to disentangle specific effects of motor function and neuronal circuitry on behavioral outcomes. EAE mice exhibit cognitive impairment, which is commonly observed in patients with MS (Sadek et al., 2023; Rocca et al., 2018). Cognitive impairment in EAE mice could not be significantly ameliorated through the ACE treatment in this study, although a previous study has documented the protective effects of ACE treatment in improving memory and learning dysfunction in vascular dementia rat models (Zhu et al., 2023).

In MS, pro-inflammatory cytokines, such as TNF-α, IL-1α, and IL-1β, along with Th1 and Th17 cells, are upregulated (Kebir et al., 2007; Becher et al., 2017). By contrast, the levels of CD4^+^ T helper (Th) cells, particularly IL-17-secreting Th17 cells, also increase within the brain (Kebir et al., 2007; Becher et al., 2017; Moser et al., 2020). Th1 and Th17 cells represent primary pathogenic T-cell populations driving demyelination and inflammation during EAE. Conversely, Treg cells play a protective role by restraining inflammatory responses and preventing tissue injury related to MS/EAE (Stromnes et al., 2008; Jäger et al., 2009). Our results showed that the ACE treatment reduces Th1 and Th17 cell populations while increasing Treg cells in the spleen and ILNs, aligning with prior findings (Zhang et al., 2014). Th17 and Treg cell dysregulation is crucially involved in MS genesis and progression (Venkatesha et al., 2015). Several drugs used for MS treatment are known to suppress immune responses. Fingolimod, the sphingosine-1 phosphate receptor agonist, acts by retaining lymphocytes within lymph nodes, thus blocking them from entering the lymphatic system (Mehling et al., 2011). This prevents death of the human brain microvascular endothelial cells owing to the increased levels of proinflammatory cytokines (Cohen and Chun, 2011). Laquinimod, the immunomodulator linomide (quinoline-3-carboxamide) derivative, inhibits Th1 and Th17 cell migration into brain endothelial cells (Lühder et al., 2017). In this study, serum levels of TNF-α, IL-1β, and IL-6 were elevated in the EAE mice, which remarkably decreased after ACE treatment. In splenic and ILN single-cell suspensions, the types of T-cell responses, including Th1/Th17, decreased in the ACE-treated versus EAE groups, whereas the number of Treg cells increased. Consistent with the previously reported results (Zhang et al., 2014), ACE modulated immune responses associated with inflammatory disease occurrence and development. In the present study, transcriptomic sequencing of mouse spinal cords demonstrated that DEGs in the EAE-ACE versus EAE groups were enriched in inflammatory response-related processes.

Microglial cells and astrocytes represent common CNS immunocytes, and their increase is crucial for MS (Distéfano-Gagné et al., 2023). Neurotoxic reactive astrocytes can kill oligodendrocytes and neurons through saturated lipids, thereby upregulating numerous classical complement cascade genes to disrupt synapses (Guttenplan et al., 2021). Astrocytes are activated in many human neurodegenerative and neuroinflammatory disorders. They are a key for the entry of peripheral immunocytes into the CNS, being active within MS lesions (Ponath et al., 2018). According to our results, EAE mice had markedly elevated microglial cells and reactive astrocytes, both of which decreased after ACE treatment, although the specific underlying mechanisms need to be elucidated. We also examined the top 20 KEGG enriched pathways. The MAPK pathway has three key subfamilies, namely p38, JNK, and ERK MAPK, with ERK being the most classical pathway regulating downstream inflammatory cytokine levels (Qi and Elion, 2005). Additionally, the AKT signaling pathway is involved in inflammation, autoimmune diseases, and neurodegenerative diseases (Manning and Toker, 2017). According to previous results from a CFA-mediated inflammatory pain rat model, ACE resisted hyperalgesia through p38 MAPK, rather than ERK (Du et al., 2017). However, our results indicated that among the KEGG enriched pathways, the ERK and JNK pathways are potentially suppressed by the ACE treatment, which explain its main action mechanism. The specific signaling pathways regulated by ACE in EAE need to be further explored.

Our study results demonstrated that ACE protects against EAE. Notably, ACE alleviates neuroinflammation, concurrently enhancing the emotional and behavioral responses. This implies the multifaceted role of ACE, which involves both peripheral and central mechanisms, thereby affecting various cellular components. The multi-targeting and multi-therapeutic characteristics of ACE need to be explored further. Despite these promising results, our study has limitations. Initially, the precise action mechanisms of ACE in exerting the protective effects remain to be fully clarified. Further exploration of related pathways, such as the JNK and ERK pathways, and the execution of rescue experiments are needed for confirming the aforementioned hypotheses. Importantly, glial activation is complex and may involve multiple signaling cascades. The interaction between these pathways and the effects of ACE on glial cells warrant further investigation. Additionally, the EAE model does not completely replicate all clinical, pathological, or immunological characteristics of MS in humans (Robinson et al., 2014). Consequently, the efficacy and safety of ACE in human MS patients should be examined through comprehensive clinical trials. In conclusion, additional mechanistic studies must be conducted to understand how ACE’s multifaceted effects contribute to its therapeutic efficacy.

## 5 Conclusion

The present study confirmed that ACE protected against EAE by modulating the cytokine levels and T-cell responses. Additionally, ACE’s effects on EAE are tightly associated with microglial and astrocytic activation, which may be attributed to its regulatory role in the ERK and JNK signaling pathways. This study supports that ACE exerts therapeutic effects on autoimmune neuroimmunology by acting on multiple targets, which offers clues for exploring the potential effects and specific mechanisms of TCMs in the treatment of CNS disorders.

## Data Availability

The RNA-seq data are accessible in the BioProject PRJNA1200241 (SRR31781072 - SRR31781083) of the Sequence Read Archive (SRA) database (https://www.ncbi.nlm.nih.gov/bioproject/PRJNA1200241).

## Footnotes

^1^ https://BioRender.com/b76k751

^2^ https://www.omicstudio.cn/tool/6
