# Supplementary material for: Acupoint catgut embedding alleviates experimental autoimmune encephalomyelitis by modulating neuroinflammation and potentially inhibiting glia activation through JNK and ERK pathways
Source: Front Neurosci. 2025 Jan 9;18:1520092. doi: 10.3389/fnins.2024.1520092 (PMC11755674; doi:10.3389/fnins.2024.1520092)
Supplement: Supplementary file 1 [file Data_Sheet_1.docx]

**Supplemental Table 1A. TH1/TH17 panel**

| Antigen | Fluorescence | Catalog numbers | Manufacturers |
| --- | --- | --- | --- |
| CD4 | FITC | 100406 | Biolegend |
| IL-17A | PE | 12-7177-81 | Invitrogen |
| INF-γ | PerCP/Cy5.5 | 45-7311-82 | Invitrogen |
| Zombie AquaTM | NUV525 | 423101 | Biolegend |

**Supplemental Table 1B. Treg panel**

| Antigen | Fluorescence | Catalog numbers | Manufacturers |
| --- | --- | --- | --- |
| CD4 | FITC | 100406 | Biolegend |
| Zombie AquaTM | NUV525 | 423101 | Biolegend |
| CD25 | PE/CY7 | 102016 | Biolegend |
| FOXP3 | PE | 12-5773-82 | Invitrogen |

| Name of primers | Sequence of primers |
| --- | --- |
| GAPDH for | CCTGGAGAAACCTGCCAAGTA |
| GAPDH reverse | ACCAGGAAATGAGCTTGACA |
| C3 for | CCAGCTCCCCATTAGCTCTG |
| C3 reverse | GCACTTGCCTCTTTAGGAAGTC |
| S100A10 for | CCTCTGGCTGTGGACAAAAT |
| S100A10 reverse | CTGCTCACAAGAAGCAGTGG |
| iNOS for | CTTGCCACGGACGAGAC |
| iNOS reverse | TCATTGTACTCTGAGGGCTGA |
| IL-10 for | ATGCTTTCTGGACTGCTGCC |
| IL-10 reverse | ATTGTTCCTGCGCCAAGAGG |

**Supplemental Table 2. List of primers used in this paper**

**Supplemental Figure 1**

**
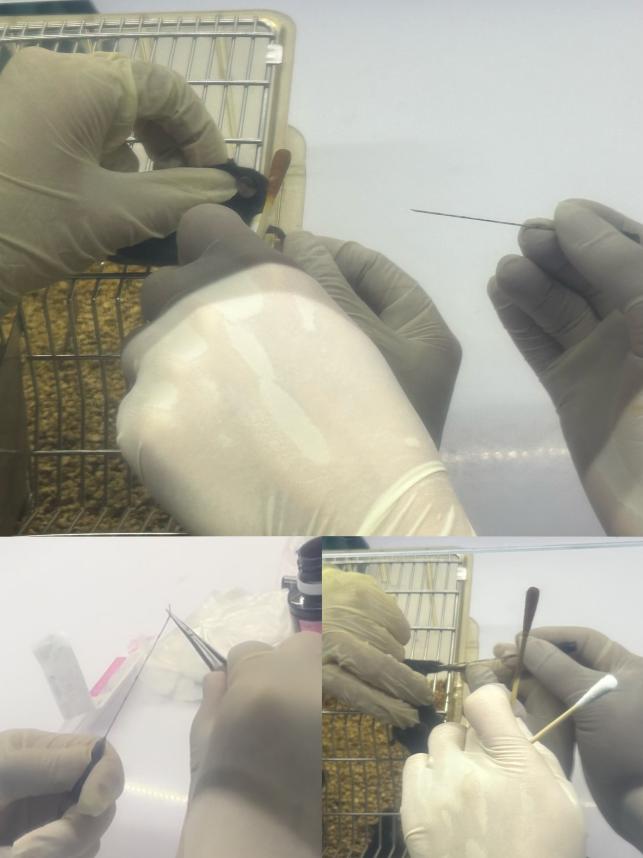
**

**Images of the mice during the ACE process**

**Supplemental Figure 2**

**
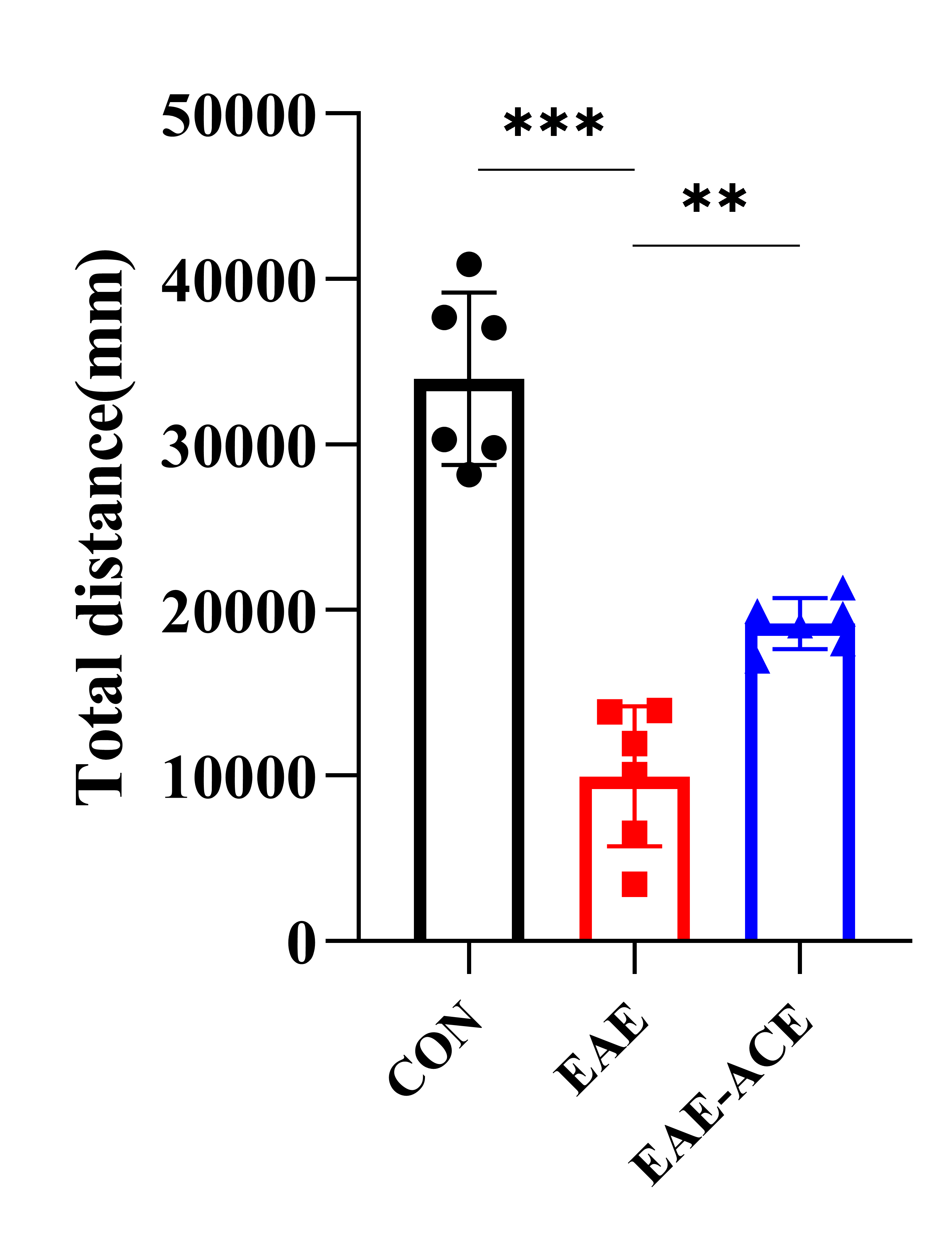

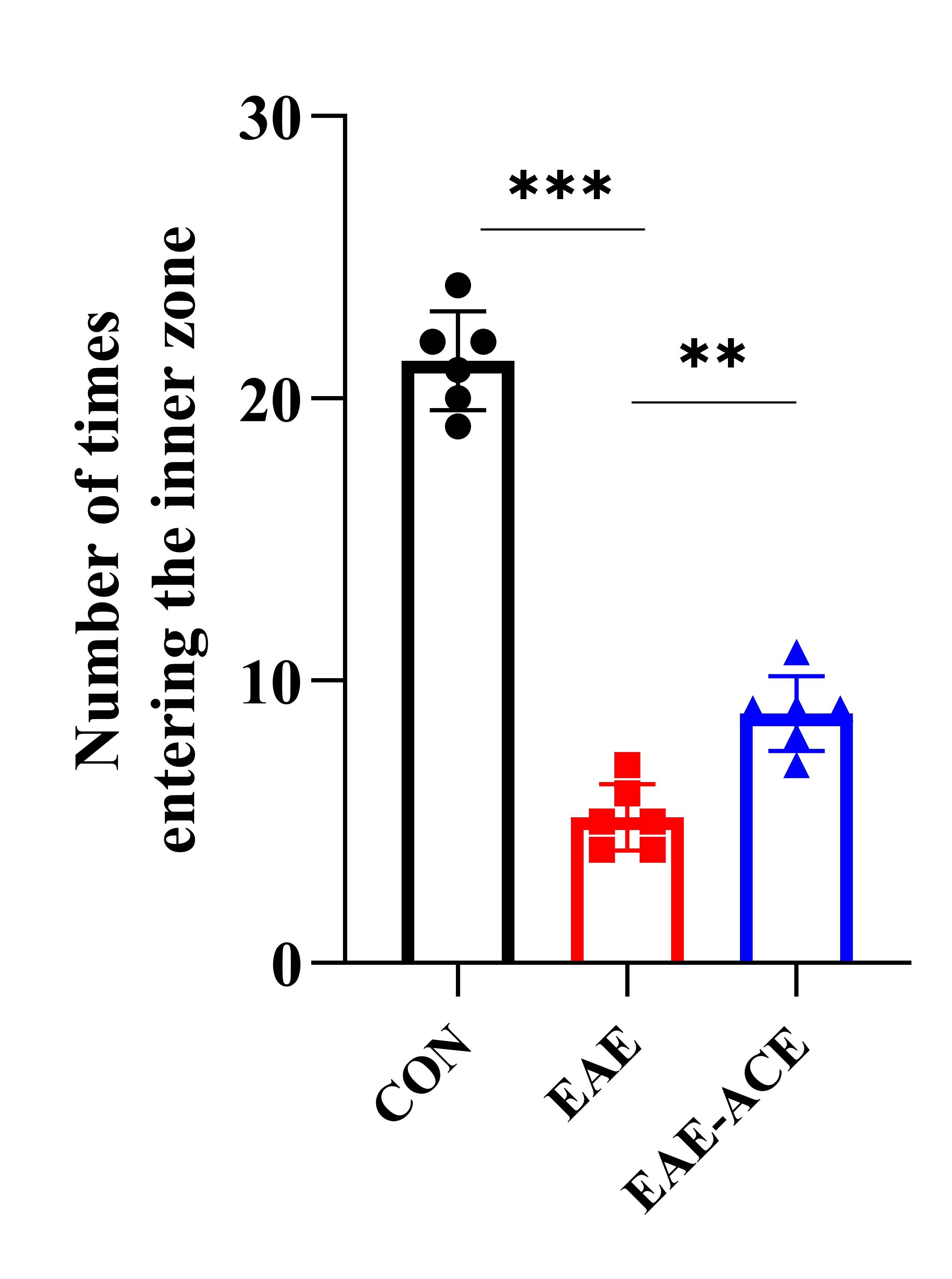
**

In OFT, the number of entries into the inner zone and total travel distance decreased in EAE mice, but increased significantly after ACE treatment.

**Supplemental Figure 3**

**
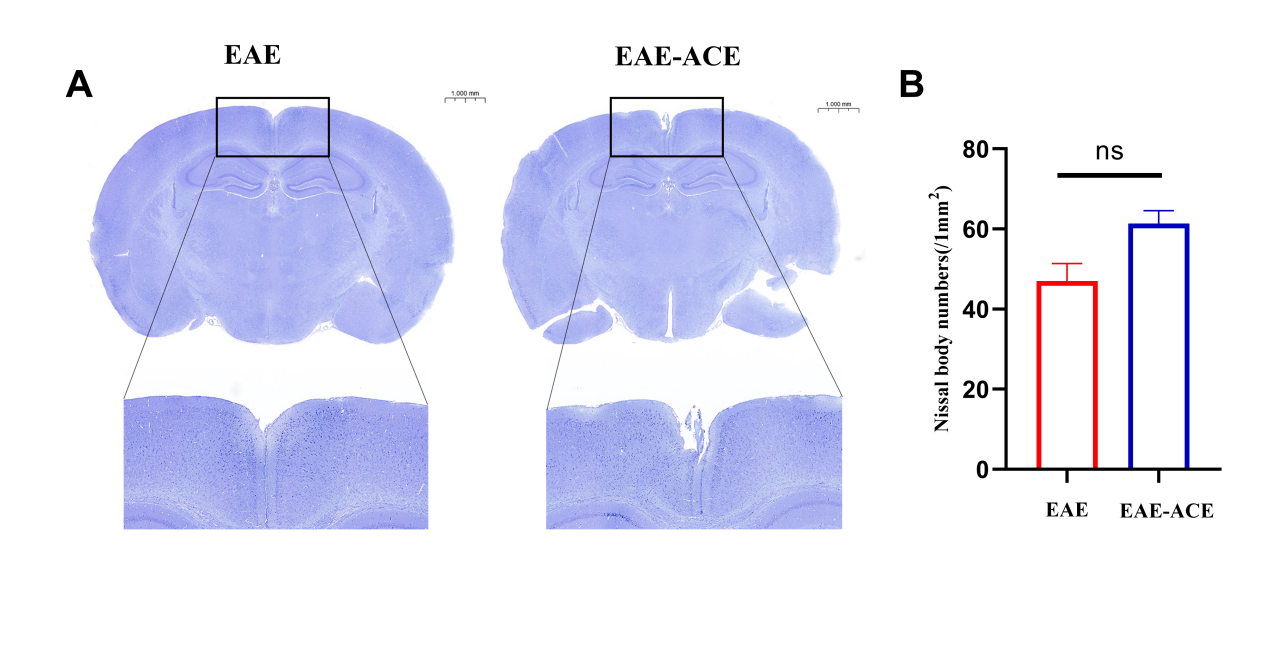
**

**Supplemental Fig.3. Effect of ACE on Nissl staining of brain tissue in EAE mice. At 21 d post immunization, brain was isolated and performed Nissl staining.** (A) Nissl staining in the brain of EAE and EAE-ACE mice. Scale bars, 50 1000mm. (B) Quantification of the nissal body of brain as shown in (A) (n = 3, Student’s t-test).

**Supplemental Figure4**

**
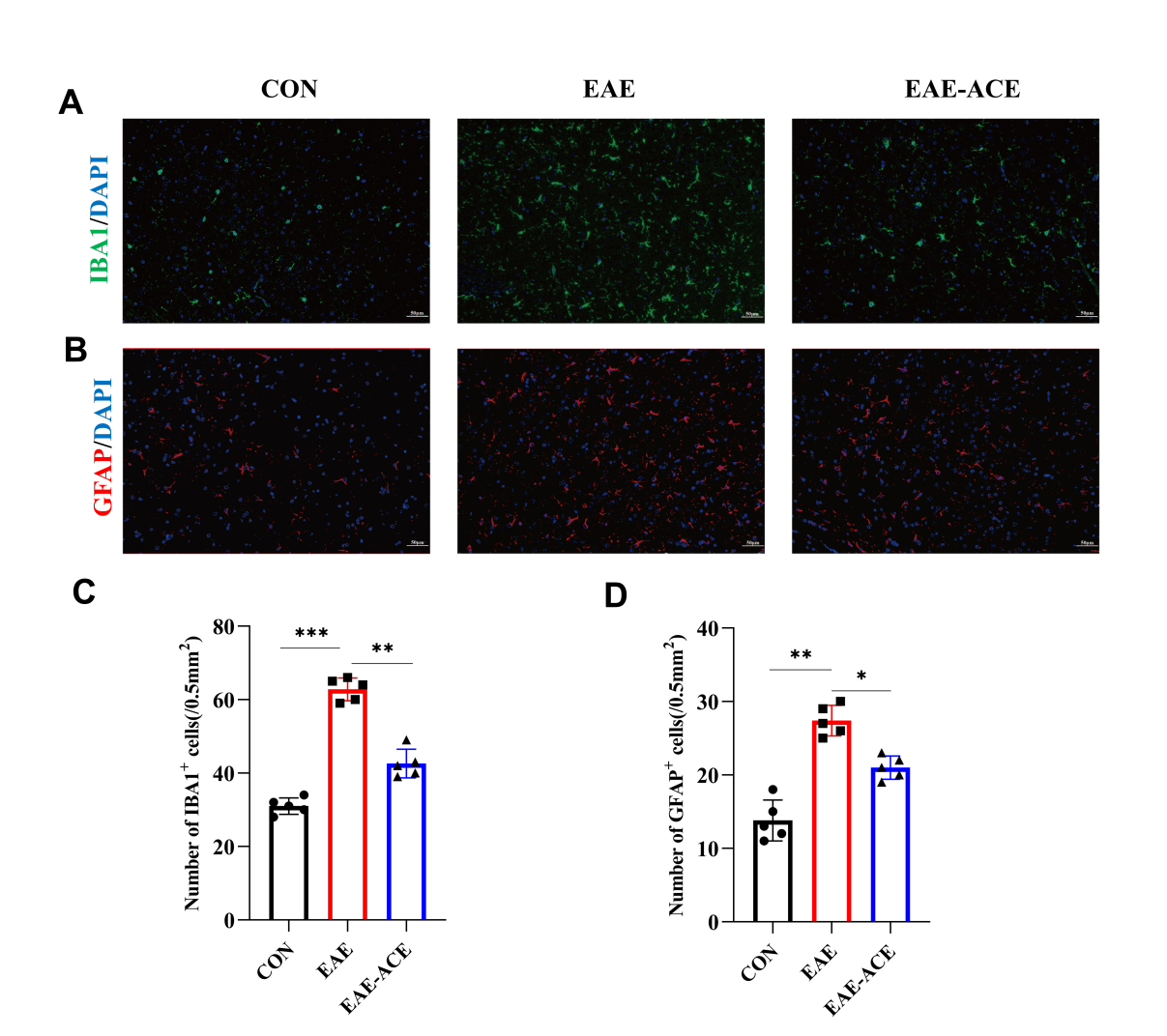
**

**Supplemental Fig.4. Immunohistochemistry analysis of microglia and astrocyte in the cortex.**

(A, B) Microglial reactivity, indicated by Iba1 antibody staining, and astrocyte reactivity, indicated by GFAP antibody staining, were assessed in the cortex of control, EAE, and EAE-ACE mice. Iba1+ and GFAP+ cells in the cortex were quantified using ImageJ software (C, D). n = 5. Scale bar,50μm.Results are presented as the mean ± SD. **p* < 0.05, ***p*< 0.01, ****p*< 0.001, one-way ANOVA with Bonferroni’s post-tests.
