# Supplementary material for: Acupoint catgut embedding alleviates experimental autoimmune encephalomyelitis by modulating neuroinflammation and potentially inhibiting glia activation through JNK and ERK pathways
Source: Front Neurosci. 2025 Jan 9;18:1520092. doi: 10.3389/fnins.2024.1520092 (PMC11755674; doi:10.3389/fnins.2024.1520092)
Supplement: Supplementary file 2 [file Presentation_1.pdf]

☆标准研制☆

实验动物常用穴位名称与定位 第3部分:小鼠

中国针灸学会

本文节选自团体标准T/CAAM 0002-2020《实验动物常用穴位名称与定位 第3部分:小鼠》,2020年5月15日由中国针灸学会发布,2020年10月31日开始实施。负责起草单位:中国中医科学院针灸研究所、南京中医药大学。主要起草人:景向红、华兴邦。参与起草人:白万柱、徐斌、徐东升、郭义、马铁明、王欣君、卢圣锋。出版发行单位:中国标准出版社。该标准以1992年江苏省中医研究所李辞蓉、华兴邦等绘制的小鼠穴位图谱为基础,修订了其中部分穴位名称及定位等表述,穴位数量较之前新增20个,删减未与人体腧穴相对应的穴位1个。规定了实验小鼠常用39个穴位的名称、定位、局部解剖以及图谱。适用于C3H/C58/C57L/A/AKR//RF/SWR等各品系实验用小鼠。本文节选小鼠常用穴位名称与定位,并附图陈列如下。

7.1 头颈部穴

7.1.1

水沟(GV 26)

定位:鼻尖下正中处。

7.1.2

百会(GV 20)

定位:顶骨正中。

7.1.3

风府(GV 16)

定位:枕骨顶嵴后枕寰关节背凹陷处。

7.1.4

耳尖(EX-HN 6)

定位:耳尖后缘。

7.1.5

承浆(CV 24)

定位:下唇毛际下1 mm。

7.2 胸腹部穴

7.2.1

膻中(CV 17)

定位:腹正中线上,平第4肋间。

7.2.2

中脘(CV 12)

定位:脐与剑突尖端连线中点。

7.2.3

神阙(CV 8)

定位:脐中央。

7.2.4

关元(CV 4)

定位:脐下约10 mm处。

7.3 背部穴

7.3.1

大椎(GV 14)

定位:第7颈椎与第1胸椎间,背部正中。

7.3.2

肺俞(BL 13)

定位:第3胸椎下两旁肋间,背正中线旁开3 mm。

7.3.3

心俞(BL 15)

定位:第5胸椎下两旁肋间,背正中线旁开3 mm。

7.3.4

膈俞(BL 17)

定位:第7胸椎下两旁肋间,背正中线旁开3 mm。

7.3.5

脊中(GV 6)

定位:在第11与第12胸椎棘突间。

7.3.6

脾俞(BL 20)

定位:第12胸椎下两旁肋间,背正中线旁开3 mm。

7.3.7

命门(GV 4)

定位:背正中线上,第2腰椎棘突下凹陷处。

7.3.8

肾俞(BL 23)

定位:在第2腰椎下两旁,背正中线旁开3 mm。

7.4 前肢穴

7.4.1

曲池(LI 11)

定位:桡骨近端的关节外侧前方的凹陷中。

7.4.2

尺泽(LU 5)

定位:在肘弯横纹偏外的凹陷中。

7.4.3

少海(HT 3)

定位:前肢肘关节内侧横纹与肱骨髁间凹陷中。

7.4.4

手三里(LI 10)

定位:桡骨前缘,前臂上1/4处。

7.4.5

内关(PC 6)

定位:前肢内侧,离腕关节约2 mm的桡尺骨缝间。

7.4.6

外关(TE 5)

定位:前肢外侧,距腕关节约2 mm左右的尺桡骨间。

7.4.7

神门(HT 7)

(C)1994-2022 China Academic Journal Electronic Publishing House. All rights reserved. http://www.cnki.net

定位：前肢内侧腕部横纹尺骨边缘。

7.4.8

太渊（LU 9）

定位：腕横纹之桡侧凹陷处。

7.4.9

合谷（LI 4）

定位：前肢第 1 与第 2 掌骨之间。

7.4.10

后溪(SI 3)

定位：第 5 掌骨头后方掌横纹头。

7.4.11

八邪（EX-UE 9）

定位：前肢第 1-4 指间，指蹼缘后方赤白肉际处。

7.5 后肢穴

7.5.1

环跳（GB 30）

定位：后肢髋关节后上缘。

7.5.2

阳陵泉（GB 34）

定位：小腿外侧，在腓骨头前下方凹陷处，距足三里上外侧约 2 mm。

7.5.3

足三里（ST 36）

定位：膝关节后外侧，在腓骨小头下约 2 mm 处。

7.5.4

三阴交（SP 6）

定位：后肢内踝尖直上约 5 mm。

7.5.5

昆仑(BL 60)

定位：后肢外踝与跟腱之间的凹陷中。

7.5.6

照海(KI 6)

定位：后肢内踝下 1 mm。

7.5.7

申脉（BL 62）

定位：后肢外踝正下方凹陷中。

7.5.8

太冲（LR 3）

定位：后肢足背第 1 与第 2 跖骨间凹陷处。

7.5.9

八风（EX-LE 10）

定位：后肢足背第 1-5 跖趾关节后缘。

7.5.10

涌泉（KI 1）

定位：后足掌心前正中。

7.6 尾部穴

7.6.1

长强（GV 1）

定位：尾根与肛门之间的凹陷处。

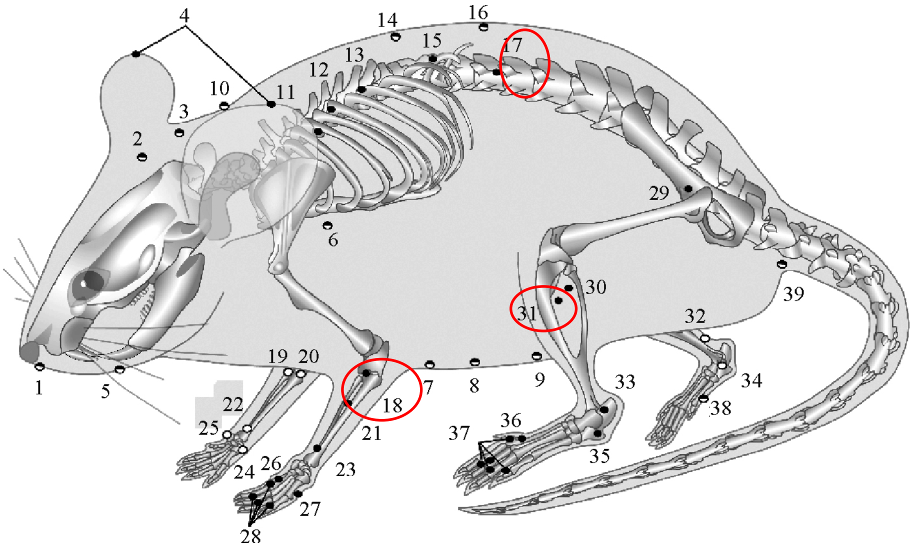

注：1/2/3 等穴位编号仅作为本图谱的标注发挥示意说明作用。

●表示在外侧面　◐表示在背腹/四肢中线　○表示在内侧面

1. 水沟　2. 百会　3. 风府　4. 耳尖　5. 承浆　6. 膻中　7. 中脘　8. 神阙　9. 关元　10. 大椎　11. 肺俞　12. 心俞　13. 膈俞　14. 脊中　15. 脾俞　16. 命门　17. 肾俞　18. 曲池　19. 尺泽　20. 少海　21. 手三里　22. 内关　23. 外关　24. 神门　25. 太渊　26. 合谷　27. 后溪　28. 八邪　29. 环跳　30. 阳陵泉　31. 足三里　32. 三阴交　33. 昆仑　34. 照海　35. 申脉　36. 太冲　37. 八风　38. 涌泉　39. 长强

(C)1994-2022 China Academic Journal Electronic Publishing House. All rights reserved.　http://www.cnki.net
